# Recognising, understanding and managing endometriosis

**DOI:** 10.4103/0974-1208.44112

**Published:** 2008

**Authors:** IAN S. FRASER

**Affiliations:** Professor Fraser is a Professor in Reproductive Medicine, Department of Obstetrics and Gynaecology, Queen Elizabeth II Research Institute for Mothers and Infants, University of Sydney, NSW

**Keywords:** Endometriosis, laparoscopy, pathological mechanisms, diagnosis, management

## Abstract

Endometriosis is defined as the presence of tissue lesions or nodules, histologically similar to the endometrium, at sites outside the uterus. It is a highly variable condition that has a wide spectrum of symptoms. The aetiology of endometriosis is probably multifactorial, with a strong familial component recognised. Women with endometriosis have multiple disturbances of function in the eutopic endometrium that women without the disease do not have. A firm diagnosis of endometriosis is rarely possible in general practice. The ‘gold standard’ for the diagnosis of pelvic endometriosis is currently a diagnostic laparoscopy.

Endometriosis is a chronic recurring disease that is often left undiagnosed. A high level of suspicion is required in women who present with pelvic pain.

‘One of the most famous sufferers from endometriosis was Marilyn Monroe. The condition was so severe that it destroyed her marriages, her wish for children, her career and ultimately her life. In days before effective conservative surgery or effective medical therapies, it led to progressively increasing use of strong analgesics, tranquilisers and hypnotics – and drug dependency.’[1]

Fortunately, few cases nowadays end like poor Marilyn's, but a minority of cases are still almost as difficult to manage. Endometriosis is a word that is now widely recognised by Australian women (indeed, widely called ‘endo’ in the community), yet it is a disease that is still not accorded as much serious attention as it ought to be by doctors. It is extremely common.[2] Specialist gynaecologists see numerous patients in whom there has been a substantial delay in diagnosis, often with classic symptoms overlooked for many years. In many developed countries the average delay between the onset of symptoms and diagnosis is still six to seven years. This can be greatly extended to up to 10 years when pain symptoms begin in the teenage years.

## DEFINITION

Endometriosis is still conventionally defined as the presence of tissue lesions or nodules that are histologically similar to the endometrium, but are present at sites outside the uterus. The incidence and prevalence of endometriosis cannot be accurately determined because of the uncertainties in making a definite diagnosis without laparoscopy. It is thought to affect up to 5 to 10% of women of reproductive age, although in those presenting with pelvic pain and/or infertility its frequency may reach 50%.

### Aetiology

The aetiology of endometriosis is still controversial and is almost certainly multifactorial.[3] A strong familial component has been recognised for many years, with a young woman who has a sister or mother with proven disease having an eightfold greater risk of developing endometriosis than a woman with no family history. This very important underlying familial predisposition is probably influenced by a multiplicity of genes with different functions, which have proven difficult to identify.[4,5]

Other factors that appear to play important roles in determining if a woman will develop the clinical condition include:

- reproductive lifestyle, especially a delay in child-bearing
- poorly understood immunological factors
- some environmental factors, probably including exposure to a range of environmental toxins
- reproductive tract occlusion, such as an imperforate hymen.

The theory that toxic environmental substances with a weak oestrogenic effect play an important role is gradually gaining experimental support.[6]

### Mechanisms of development

The most widely accepted mechanism by which endometriosis develops is through retrograde menstruation of viable fragments of endometrium, which are then able to implant on the peritoneal surface.^7^ However, this cannot be the only mechanism of development, because retrograde menstruation occurs in almost all women and most do not develop the clinical features of endometriosis. It seems probable that there needs to be abnormalities of function, either within the eutopic endometrium lining the uterus of women predisposed to endometriosis and/or impaired immune surveillance mechanisms normally responsible for the recognition and removal of endometrial fragments, which find their way into the peritoneal cavity.

Evidence to support this theory includes the observed distribution of the most common peritoneal endometriotic lesions close to the fimbrial ends of the fallopian tubes on the broad ligaments, the uterosacral ligaments, the pouch of Douglas and the surface of the ovaries [Table 1]. Also, endometrial cells recovered from the pelvic cavity at the end of menstruation are viable and capable of proliferating. These same endometrial cells express adhesion molecules that allow them to attach to the peritoneal surface. They may also express the aromatase enzyme (allowing them to synthesise active local oestrogen) and have the capacity to secrete angiogenic and neurogenic molecules (encouraging tissue growth and potential pain-generation mechanisms within the developing lesions).

**Table 1:** Anatomical sites for development of endometriosis

Feature of endometriosis	Anatomical site where found
Deposits on pelvic peritoneal surfaces*	Uterosacral ligaments
	Pouch of Douglas
	Broad ligaments
	Ovarian surfaces
	Bladder peritoneum
Deep ovarian deposits*	Ovarian endometriomas (‘chocolate cysts’) – deep invaginations into the ovarian surface, with cystic contents of altered blood. These cystic structures are usually adherent to the underlying broad ligament
Deep pelvic nodules† (usually dense, partially fibrous lesions; histology may have similarities to adenomyosis)	Rectovaginal septum
	Vaginal vault
	Sometimes involving the rectal wall
Lesions involving other abdominal organs†	Bowel – surface of rectum or sigmoid colon
	Tip of appendix
	Bladder
	Ureter
	Diaphragm
	Umbilicus
	Sciatic nerve roots
Distant lesions‡	Pleura
	Lung
	Brain
	Other sites

* Common

† uncommon

‡ rare

A novel concept developed by Australian researchers proposes that women who develop endometriosis are shedding endometrial stem cells from basal endometrium during menstruation. These rare cells (only present in very small numbers in the endometrium) have the unique ability to produce progenitor cells, which in turn have a great capacity for proliferation and perhaps the potential to initiate a lesion from a single cell.

### Endometriosis lesions

Peritoneal lesions mature through a series of stages from clear surface papules to ‘red flare’ lesions with active new blood vessel formation [Figure 1A and B]. They then progress to white scarred lesions and mature ‘black’ lesions containing inspissated old blood deposits under the peritoneal surface. This progression is calculated to take about 10 years. Endometriomas containing large volumes of altered menstrual blood products are usually only seen within the ovaries [Figure 2] and can grow to more than 10 cm in diameter. These are actually pseudocysts, which invaginate into the surface of the ovary from adherent lesions on the pelvic sidewall. Deep pelvic nodules of endometriosis may develop in the rectovaginal septum. These tend to contain a great deal of fibrous tissue and have similar histological appearances to adenomyosis. There is controversy as to whether these different types of endometriosis actually constitute different forms of the disease or whether they are merely the result of accidental development of the ectopic deposits in different pelvic sites.

**Figure 1A:**
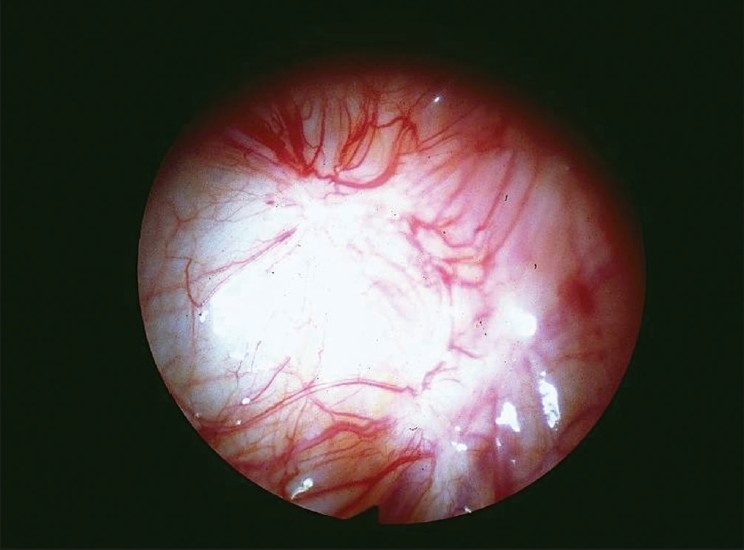
Peritoneal lesion of classical endometriosis showing a ‘redflare’ of new blood vessels illustrating angiogenesis around a scarred white deposit (courtesy of Professor Robert Jansen)

**Figure 1B:**
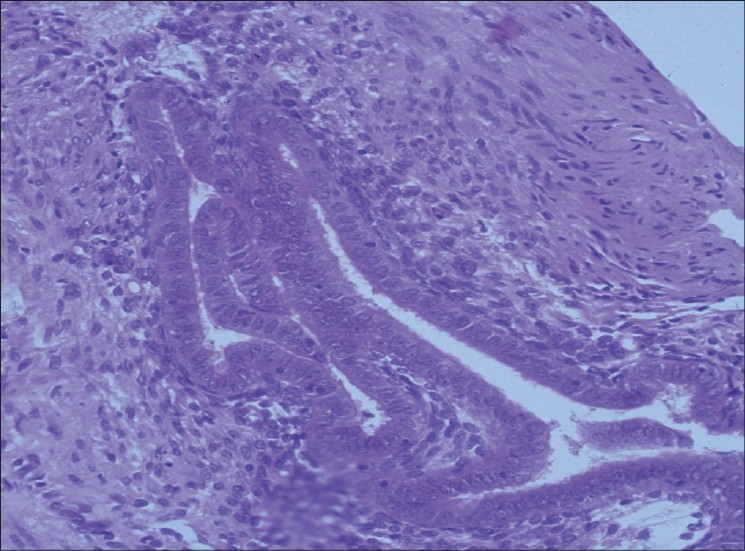
Typical histological picture of endometriotic glands and stroma below the peritoneal surface (biopsied from the lesion in Figure 1A)

**Figure 2:**
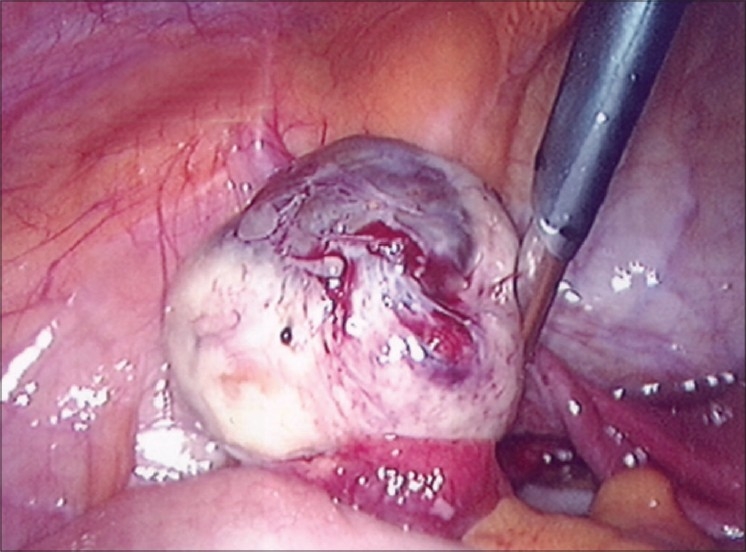
Left ovarian endometrioma after mobilisation from pelvic sidewall

### The eutopic endometrium

It is now clear that women with endometriosis have multiple disturbances of function in the eutopic endometrium (the normally situated endometrium inside the uterus) that women without the disease do not have.[8] These functional disturbances are currently being studied using a range of powerful laboratory techniques including genomics, proteomics and immunohistochemistry.

This research has so far revealed that there are major changes in the presence and function of:

- many types of endometrial immune cells capable of regulating multiple key metabolic processes
- a wide range of endometrial proteins [Table 2] including structural cell proteins – for example, cytokeratins, vimentin and actin
- apoptosis (the process of organised or ‘programmed’ cell death)
- cellular adhesion molecules, growth factors, signalling molecules and aromatase enzymes
- angiogenesis (the process of new blood vessel formation)
- lymphangiogenesis (new lymph vessel formation)
- neurogenesis (the growth of new nerve fibres).

**Table 2:** Probable complex pathological mechanisms underlying endometriosis

Probable primary anomalies of endometrial function	
•	Increased intrinsic aromatase enzyme activity, resulting in local oestrogen production
•	Reduced apoptosis, resulting in greater viability of menstrually shed tissue fragments
•	Increased production of intercellular adhesion molecules, resulting in an increased likelihood of cells attaching to the peritoneum
•	Increased secretion of angiogenic molecules, such as vascular endothelial growth factors, which stimulate the growth of new blood vessels and lymph vessels
•	Decreased response of endometrial mechanisms to progesterone stimulation
•	Greatly increased secretion of neurogenic molecules, such as nerve growth factor and nerve growth factor receptor, which stimulate the development of new sensory nerves within the endometrium, myometrium and endometriotic lesions
•	Several changes in immune cell populations, such as macrophages, dendritic cells, plasma cells and their associated secretory products, including cytokines and many related molecules
•	Anomalies have been shown in many other molecular systems, such as the generation of free oxygen radicals, structural proteins, many growth factors and signalling molecules
Probable anomalies within the peritoneum, peritoneal fluid and ectopic lesions	
•	Increased numbers of macrophages, but they appear to have reduced capability to 'mop up' cell fragments
•	Disturbance of other immune cells, such as dendritic cells (decreased) and plasma cells (increased)
•	Increased expression of many molecules, such as prostaglandins, which have the potential to stimulate sensory nerve fibres
•	Many molecular systems reflecting the functional anomalies seen in eutopic endometrium
Differences between endometriotic lesion and eutopic endometrium functions	
•	There are many differences between endometrium from women without endometriosis and the function of endometriotic lesions, as could be predicted from the list of functional anomalies seen in endometrium from women with endometriosis. However there also appear to be subtle differences between the function of lesions and function of the eutopic endometrium from which they arose
•	These subtleties of functional difference are currently under intense study, but one obvious difference is that hormonal therapy almost completely eliminates nerve fibres from the functional layer of eutopic endometrium, but it does not eliminate them from ectopic lesions

There also seems to be a general reduction in response of the tissue to stimulation by progesterone.

Our latest research from the University of Sydney has revealed that fine sensory nerve fibres are present throughout the functional layer of endometrium in all women with endometriosis, but not found in those without endometriosis.[9] The density of nerve fibres in the myometrium is increased and a high concentration of nerve fibres are also present in the ectopic peritoneal endometriosis lesions. These nerve fibres must play some role in pain-generating mechanisms.

Following demonstration of these multiple functional and microanatomical abnormalities within eutopic endometrium, with no obvious single common underlying molecular abnormality, it is hypothesised that endometriosis is an example of a disease where multigene activation occurs. The presence of multiple genes modulated by many co-activators and co-repressors could help to explain the great variability of endometriosis.

This theory does not explain the cause of all cases of endometriosis or fully explain the implantation theory. In some situations there is evidence to support the involvement of coelomic metaplasia, development from embryonic rests (a fragment of embryonic tissue that has been retained after the period of embryonic development), lymphatic and vascular metastases and indeed a combination of some or all of these theories. Variations in pathogenesis may explain the development of endometriosis in other anatomical sites [Table 1].

There is also increasing evidence to support the hypothesis that endometriosis is primarily an ‘endometrial’ disease. The many endometrial functional disturbances described appear to occur before the development of ectopic lesions and these key changes in the endometrium of women with endometriosis appear essential for attachment, viability, growth and innervation of fragments of menstrually-shed endometrium in ectopic sites. The key functional anomalies appear to be the expression of intercellular adhesion molecules, the presence of local aromatase enzyme activity, decreased apoptosis, increased angiogenesis and increased neurogenesis [Table 2].

### Symptom variability

Endometriosis is a highly variable condition that has a wide spectrum in the experience of symptoms, age of presentation, anatomical extent of disease, incidence of infertility, response to treatment, likelihood of recurrence, rate of progression and natural long-term history of the condition. Indeed, a small minority of women will exhibit this condition without experiencing any symptoms. It is unclear if this great variability is genetically predetermined or is influenced by exposure to lifestyle or environmental factors.

It seems that the great variability of the condition is partly responsible for the considerable delay in diagnosis that often occurs. Sadly, this delay still occurs in many women who present with the classic symptoms of considerable dysmenorrhoea or chronic pelvic pain. A high index of suspicion should be maintained in all women with pelvic pain, even when the presentation may be atypical, because although the spectrum of symptoms is highly variable, the great majority of women with significant endometriosis will present with some form of pelvic pain [Table 3]. A minority will not be aware of any substantial symptoms or may complain solely of infertility.

**Table 3:** Symptoms of endometriosis*

•	Congestive dragging dysmenorrhoea and backache, beginning well before onset of menses
•	Deep dyspareunia and secondary loss of libido or vaginismus
•	Premenstrual spotting, heavy menstrual bleeding, intermenstrual bleeding, menstruation prolonged beyond eight days
•	Major lethargy just before and during menses
•	Pain with bowel movement, tenesmus or rectal bleeding during menstruation
•	Perimenstrual diarrhoea and/or constipation and troublesome painful abdominal bloating, often mimicking irritable bowel syndrome but not relieved by a bowel motion
•	Generalised pelvic discomfort; erratic, sudden acute, sharp or dull pelvic pain; peri-ovulatory pain
•	Urinary urgency or bladder discomfort, pain with urination, rarely haematuria
•	Other vicarious menstruation (e.g. haemoptysis); rare
•	Infertility

* Many of these symptoms are 'classic' and well recognised and should not be overlooked. However, the combination of presenting symptoms can be highly variable and a high index of suspicion is needed.

The majority of women with some form of pelvic pain will also report disturbances of uterine bleeding, multiple bowel symptoms (often misdiagnosed as irritable bowel syndrome), painful abdominal bloating, cyclical urinary symptoms, extreme cyclical lethargy or occasionally very unusual symptoms [Table 3]. The progress of the condition is also unpredictable.

### Natural history of the condition

The natural history of endometriosis is poorly understood, because few patients will progress through their reproductive lifespan without attempts at effective therapy.[10] In a small proportion of patients symptoms will resolve spontaneously, but in most the symptoms will persist or worsen. Some women appear to be ‘cured’ of the condition after effective surgery, but most will experience a recurrence of symptoms. Endometriosis usually becomes inactive after the menopause and few women will continue to experience symptoms beyond this time. The very occasional woman who experiences ongoing symptoms after the menopause appears to have nodules of endometriosis that are capable of producing local oestrogen through activity of the aromatase enzyme.

### Special features of adolescent endometriosis

Endometriosis appears to begin in adolescence more frequently than generally recognised and there is often an inordinate delay before a diagnosis is made (10 years or more). These girls typically have a strong family history. Menstrual pain often begins at or soon after the menarche and may initially be typical of severe primary spasmodic dysmenorrhoea beginning on about day one of menses and then gradually developing the more typical combinations of endometriosis symptoms.

Our belief is that these young women already have the genetically determined abnormalities of endometrial function that lead to the generation of pain within the endometrium and myometrium. As the typical peritoneal or ovarian lesions of endometriosis develop, innervation of these lesions rapidly follows and pain generation then begins from more distant parts of the pelvic cavity, such as the uterus. This leads to the more ‘congestive’, dragging and gnawing diffuse pelvic pain and backache of classic endometriosis, which typically begins well before menstruation.

### The challenges of diagnosis

A firm diagnosis of endometriosis is rarely possible in general practice, but a high index of suspicion can be applied. Features that may lead to specialist referral are summarised in the Annex 1. Several international research groups are currently attempting to develop simple symptomatic screening algorithms or diagnostic decision tools that will assist in the diagnosis of endometriosis in general practice. The ‘gold standard’ for the diagnosis of pelvic endometriosis is currently a diagnostic laparoscopy (and often a peritoneal biopsy) by an experienced gynaecologist for confirmation or exclusion.[11] Even this presents difficulties such as the atypical appearances of peritoneal lesions (particularly in adolescents) or deep and ‘partially hidden’ lesions, especially for the inexperienced surgeon.

**Annex 1:** When to refer a patient with endometriosis

•	‘Typical’ symptoms of congestive, dragging dysmenorrhoea with other pelvic pain symptoms (deep dyspareunia, pain with a bowel motion or ‘ovulation’ pain) which interfere with lifestyle.
•	Severe dysmenorrhoea of any pattern unresponsive or poorly responsive to several courses of different standard analgesics (paracetamol or prostaglandin inhibitors).
•	Severe or increasing localised or positional deep dyspareunia or tenderness at the vaginal vault or on cervical excitation at vaginal examination).
•	Pelvic pain in a woman with a moderate or strong family history of endometriosis.
•	Infertility of more than one year duration, especially when the woman is over 30 years and sooner when some dysmenorrhoea is present.
•	The presence of the following symptoms in someone with pelvic pain: menstrual bleeding prolonged to more than eight days; painful, cyclical abdominal bloating (with or without other gastrointestinal or ‘irritable bowel’ type symptoms, including cyclical diarrhoea and constipation); pain with urination.

Transvaginal ultrasound may make a strong presumptive diagnosis of an ovarian endometrioma, but not of other types of endometriosis and other imaging techniques are rarely helpful.

Much recent energy has been invested into research to find a specific blood test for endometriosis but with limited success. Genomic chip technologies and proteomic techniques hold considerable future promise, although they are likely to be expensive at first. We have developed a highly promising endometrial biopsy technique looking for unmyelinated nerve fibres, which has an extremely high specificity and sensitivity (almost 100%), equivalent to that of diagnostic laparoscopy.[12] Its potential use in routine clinical practice requires much more research.

### The role of imaging

Transvaginal ultrasound carried out by operators with specialised experience in the assessment of female pelvic disorders is now the mainstay of female pelvic imaging and remarkable structural and vascular detail can often be detected. Nevertheless, it is important to understand that ultrasound will generally only demonstrate endometriotic lesions, which form cysts or endometriomas in the ovaries. Peritoneal lesions cannot be demonstrated and, in most cases, neither can deep pelvic nodules in the rectovaginal septum without specialised techniques.

Ovarian endometriomas usually give a typical ‘echogenic’ signature on ultrasound but haemorrhagic corpus luteum cysts, which are very common, can mimic endometriomas. A repeat ultrasound in two or six weeks to avoid the next luteal phase may be necessary to confirm that the ‘haemorrhagic cyst’ is indeed a persistent endometrioma.

### Active management

Increasingly, the effective management of endometriosis requires an individual approach for each affected woman, because of the great variability of the disease.[11] The Cochrane Collaboration's approach to systematically reviewing the highest quality randomised trials is important when determining the primary management of women with endometriosis, but may be difficult to apply in the many women in whom initial therapies are ineffective or in whom recurrences occur.

Initial effective management generally requires the major pain symptoms to be controlled using standard analgesics, followed by surgical excision of the lesions or medical (usually hormonal) suppression of the lesion's activity. Some of the many possible therapies are listed in Tables 4 and 5.

**Table 4:** Analgesic management of endometriosis

Medication		Mode of action	
Initial analgesia (from onset of strong pain)			
•	Intensive paracetamol 500 mg tds three times daily	•	Analgesic, antipyretic (no anti-inflammatory activity); mainly central action
•	Intensive prostaglandin inhibitors – e.g. NSAIDs, mefenamic acid (Ponstan), naproxen sodium (Aleve, Anaprox, Crysanal, Naprogesic, Nurolasts), ibuprofen (e.g. Nurofen; initially two tablets or capsules; then one to two tablets or capsules every six to eight hours for three to four days; with food)	•	Inhibition of the pain-mediating effects of peripheral prostaglandins; anti-inflammatory
•	Dextropropoxyphene and paracetamol (Capadex, Di-Gesic, Paradex)	•	Analgesic, antipyretic
Stronger analgesia			
•	Paracetamol with codeine	•	Codeine potentiates the action of paracetamol
•	Tramadol (Durotram XR, Tramahexal SR, Tramal, Tramedo, Zydol)	•	Weak narcotic; has other actions; very rarely addictive
•	Oxycodone (Endone, OxyContin, OxyNorm, Proladone)	•	Narcotic alternative to morphine
•	Stronger pain relief: Rare use of intramuscular injections of pethidine or morphine with severe acute episodes of pain	•	Narcotics; act on central opioid receptors
Treatment of neuropathic pain			
•	Gabapentin (e.g. Neurontin)	•	Complex gamma-aminobutyric acid uptake inhibitor

**Table 5:** Hormonal therapies for endometriosis

Medication	Mode of action
Rapid, short-term hormonal suppression of disease symptoms Gonadotrophin-releasing hormone analogues	
Goserelin implants (Zoladex) and nafarelin nasal spray (Synarel) for a maximum six-month course	‘Pseudo-menopause’ to very effectively reduce ovarian oestrogen secretion for the duration of therapy. These therapies may be combined with oral progestogen as add-back therapy to alleviate side effects of hot flushes (e.g. 10 mg/day medroxyprogesterone acetate [Provera, Ralovera]; 5 mg/day norethisterone [Primolut N])
Danazol (Azol) and gestrinone (Dimetriose)	Weak, impeded androgenic hormonal environment with inhibition of endometrial growth and function; very effective but less popular now because of mild, dose-related androgenic side effects in a minority of women
Long-term hormonal suppression or prevention of symptom recurrence	
Combined oral contraceptive pill (OCP)	
Anecdotally 30 µg ethinyloestradiol plus 150 µg levonorgestrel (Levlen ED, Microgynon 30, Monofeme, Nordette) works best, but newer combined OCPs may also work well; given either cyclically or continuously	‘Pseudo-pregnancy’ with suppressed secretory changes in endometrium
Progestogens	
Oral – norethisterone 5 mg one to three times daily medroxyprogesterone acetate 10 mg one to three times daily Intramuscular depot medroxyprogesterone acetate	‘Pseudo-pregnancy’ with very marked, suppressed secretory or atrophic endometrial changes
(Depo-Provera, Depo-Ralovera)	
Levonorgestrel intrauterine system (Mirena)	Levonorgestrel intrauterine system is helpful in suppressing symptoms, but also seems effective at preventing recurrences
Subdermal etonogestrel implant (Implanon Implant)	Marked, suppressed secretory and atrophic endometrial changes
Specialist options for difficult cases	
Combination of an aromatase inhibitor (e.g. Letrozole [Femara], anastrozole [Arimidex]) with a combined OCP or a progestogen	Suppression of aromatase enzyme activity within lesions, plus progestogenic suppression of other ‘inflammatory’ activities within lesions
Combination of progestogen delivery systems (levonorgestrel intrauterine system and subdermal etonogestrel implant)	More effective suppression of both eutopic endometrium and ectopic lesions
Oral anti-oestrogen (e.g. tamoxifen [Genox, Nolvadex, Tamosin, Tamoxen] used off-label)	Effective suppression of oestrogen action
Likely future developments	
Oral or parenteral progesterone-receptor modulators	Mifepristone (not available yet for this purpose except in clinical trials) and other progesterone-receptor modulators are effective at suppressing endometrial/endometriotic proliferation and inflammatory functions; new developments are underway

Endometriosis is such a variable condition that individualisation of the approach to management is important in all but the initial approaches to active treatment. In certain circumstances, almost any surgical or medical approach may be the optimal initial management, although options always need to be discussed with the patient and their partner or family, if appropriate. This is a challenging and ongoing condition and it is always valuable to include a second person who the patient trusts in the consultations.

### Analgesic management

Analgesic management of endometriosis requires gradual progression from paracetamol to the nonsteroidal prostaglandin inhibitors (generally little to choose between the alternatives), followed by compound codeine alternatives and then intermittent tramadol (Durotram XR, Tramahexal SR, Tramal, Tramedo, Zydol) in adequate doses as dictated by the exacerbations of this condition. For exceptionally severe episodes, oxycodone (Endone, OxyContin, OxyNorm, Proladone), pethidine and morphine (Anamorph, Kapanol, MS Contin, MS Mono ordine, Sevredol) may be considered, but frequently repeated dosage should be avoided.

### Surgical excision

Initial surgery will almost always involve gynaecological laparoscopy for diagnosis, assessment and/or active surgical excision. Thorough surgical excision can be difficult and relatively few practitioners have the advanced laparoscopic surgical skills necessary for some of the more difficult excisions, although more young trainees are being mentored through these difficult learning curves. Surgery may merely involve excision of small and easily visible peritoneal lesions, but many of these lesions extend deeply under the peritoneal surface and a minority involve the rectum and structures adjacent to the rectovaginal septum. Therefore, these women may require the surgical attention of a team of experts, including colorectal or urological specialists, for safe and effective excision.

Surgical excision of ovarian endometriomas is one of the most controversial surgical approaches to the management of endometriosis. Ovarian endometriomas can be excised at laparoscopy or laparotomy, depending on the size of the pseudocyst and the skill of the surgeon, but the controversy focuses on the amount of tissue that is removed. ‘Stripping’ of the cyst wall removes some normal underlying ovarian cortex and only small areas of this tissue contain endometriosis. The main area of active endometriosis is around the site of attachment to the pelvic sidewall [Figure 2] and this needs to be fully excised. The best approach to identify and limit excision of the residual active areas inside the pseudocyst remains to be defined.

There is clear evidence that women with infertility associated with endometriosis benefit greatly from surgical excision of the disease,[13] whereas medical therapy has little beneficial effect.[14] Assisted reproductive technologies, such as IVF, are successful at overcoming infertility associated with endometriosis, although perhaps less successful than with other causes of infertility.

### Medical therapy

Medical therapy involves sound analgesic management followed by hormonal suppression at one of two levels. Highly effective suppression is provided by the gonadotrophin-releasing hormone (GnRH) analogues, especially the goserelin implants (Zoladex) given over six months, but danazol (Azol) provides very similar suppression. The side effects of both these medications are well known and include vasomotor symptoms for the GnRH analogues and mild androgenic changes for danazol and need to be addressed with counselling and regular follow up. Hirsutism or voice changes with danazol should dictate cessation of the medication.

Longer-term suppression is becoming more of an issue with the recognition that surgery does not usually offer such a long-term benefit as once believed. The progestogens offer the most effective levels of suppression, although not all patients respond well. Dosages may need to be individualised. The oral contraceptive pill may work satisfactorily as an initial approach to medical therapy, but only a minority do well. There is a gradual move to use the intrauterine (Mirena) and subdermal (Depo-Provera, Depo-Ralovera, Implanon Implant) progestogen delivery systems for their convenience and efficacy. Difficult cases may do even better with a combination of these two delivery systems,[15] but this is specialised therapy for women who fail to respond to other therapies.

### Prevention

Physicians are beginning to think about how they might be able to identify those women in whom recurrence after surgery is likely and, therefore, how they might provide effective therapy to prevent recurrence. The natural extension of this is to consider how to identify those women who are most likely to develop endometriosis and then produce and apply preventive lifestyles and therapies that may obviate or at least delay the onset of the disease process.[16]

Although there are few data in the literature to address this issue directly, it is likely that any prolonged exposure to progestogens alone or even to combined oral contraceptive preparations, may be effective in delaying, minimising or preventing the onset of symptomatic endometriosis in many women. It is hypothesised that because of its highly focused local pro-gestogen effect the levonorgestrel intrauterine system will have a dramatic role in the prevention of endometriosis and there are limited published data to support this concept.[17] A large scale multicentre trial is needed to address this concept. There is considerable optimism that in the future progesterone-receptor modulators will have an even better preventive effect, both of primary disease and recurrences.

### New research and information access on endometriosis

Endometriosis is one of the most actively researched of all benign gynaecological diseases, yet there are many unanswered questions about it. It is still an ‘enigmatic’ disease.

The World Endometriosis Society has been formed and runs a triennial World Congress on Endometriosis, the most recent of which was held in Melbourne in March 2008.[18] A remarkable number of lectures, presentations, videos, posters and live operating sessions were presented, indicative of the vast amount of research that is currently ongoing. As in most areas of medicine, Australia is contributing productively at the forefront of this research in several fields. A collaborative research group called the World Endometriosis Research Foundation is a joint initiative of the World Endometriosis Society, the American Society for Reproductive Medicine and the European Society for Human Reproduction and Embryology.[19]

Active progress is being made in understanding the aetiology and pathogenesis of endometriosis, in improving diagnostic and assessment techniques, in developing new approaches to medical therapy, in gaining confidence in the use of long-term medical therapy for the suppression and prevention of recurrence and in the application of new endoscopic excisional skills. We still have little understanding of the mechanisms of generation of pain and other symptoms and have so far been unsuccessful in developing successful screening algorithms for reliable earlier diagnosis.

## CONCLUSION

Endometriosis is a complex and highly variable disease that still challenges modern science in every aspect of presentation, symptoms, aetiology and mechanisms, diagnosis and management. Key aspects of understanding and managing endometriosis are highlighted in the Annex 2. Resources for patients are given in the box on this page.

**Annex 2:** Endometriosis: Key messages

•	Endometriosis is a highly variable condition in its age and mode of presentation, range of symptoms, anatomical sites, response to treatment and likelihood of recurrence.
•	Endometriosis is a chronic, recurring disease in many women and can have a devastating effect on lifestyle in a minority of them.
•	The aetiology of endometriosis is complex and unclear. Although a strong genetic component is common, reproductive and environmental factors are often important.
It may be primarily an ‘endometrial’ disease.	
•	Endometriosis can be asymptomatic, but usually presents as pelvic pain with or without infertility.
•	The gold standard for the diagnosis of endometriosis is still laparoscopy, but other less invasive approaches through endometrial biopsy and blood tests are being developed.
•	Medical and surgical treatments of endometriosis need to be individualised and many women will require both approaches at different times during the course of their disease.
•	A substantial proportion of women with endometriosis may gain long-term relief after thorough excisional laparoscopic surgery by an expert.
•	Effective long-term medical suppression and prevention of recurrence of endometriosis is becoming a reality.
•	Endometriosis is usually spontaneously cured at menopause, but may occasionally persist, especially during oestrogen-only hormone replacement therapy.

**Table:** Information for patients

The overall quality of medical information available through the internet has improved substantially in recent years, but is still of highly variable individual quality. Many websites, both professional and consumer, offer specific services and may therefore present a degree of bias. The following websites provide sound consumer information:	
•	The Endometriosis Association (USA) invests a substantial amount of effort and money into good-quality information and consumerorientated research – www.endometriosisassn.org
•	The Endometriosis Association of Victoria also provides information relevant to Australian women – www.qvwc.org.au/infohub/health_and_wellbeing (joint website with the Queen Victoria Women's Centre; type ‘endometriosis’ in the search box)
•	The Global Forum for Information about Endometriosis – www.endometriosis.org
•	The National Women's Health Resource Center provides a wide range of sound information – www.healthywomen.org (type ‘endometriosis corner• in the search box)
•	The Jean Hailes Foundation for Women's Health, based in Melbourne, provides excellent, sound consumer information and regular consumer forums – www.endometriosis.org.au
•	Some women respond well to the opportunity to exchange information in a ‘blog’ forum such as www.chronicbabe.com or www.howtocopewithpain.org
